# Intraductal papillary mucinous neoplasm in an annular pancreas: a case report

**DOI:** 10.1186/s40792-015-0068-7

**Published:** 2015-08-25

**Authors:** Shinichiro Kobayashi, Yukio Kamohara, Yasuhiro Nagata, Masahiro Ito, Hikaru Fujioka

**Affiliations:** Department of Surgery, National Hospital Organization Nagasaki Medical Center, Ohmura, Nagasaki Japan; Department of Pathology, National Hospital Organization Nagasaki Medical Center, Ohmura, Nagasaki Japan

**Keywords:** Intraductal papillary mucinous neoplasm, Annular pancreas, Partial pancreatic resection

## Abstract

Annular pancreas is a rare anomaly in which a ring of pancreatic tissue encircles the second portion of the duodenum. We herein report a case involving a 79-year-old Japanese man with an intraductal papillary mucinous neoplasm (IPMN) of the pancreas. Imaging studies showed that the pancreatic tissue encircled the descending part of the duodenum and that a 30-mm-diameter cystic tumor was present in the annular segment, leading to the diagnosis of pancreatic IPMN. Limited pancreatic resection was successfully performed by careful division of the annular segment from the second portion of the duodenum. The postoperative course was uneventful, and the patient’s pancreatic function was retained without the need for supplementation. To the best of our knowledge, this is the first report of IPMN occurring in the annular segment of the pancreas. Limited resection of the pancreatic annular segment is a feasible surgical treatment for noninvasive IPMN of the annular pancreas.

## Background

Annular pancreas is a rare congenital anomaly caused by malrotation of the pancreatic ventral bud during embryonic development. This condition was first reported by Tiedemann in 1818 [1]. Autopsy and intraoperative studies have estimated the incidence of annular pancreas to be approximately 5 to 15 cases per 100,000 patients [2]. The coexistence of an annular pancreas with a pancreatic neoplasm is therefore exceptionally rare.

We herein report a case of an intraductal papillary mucinous neoplasm (IPMN) in an annular pancreas.

## Case presentation

**Fig. 1:**
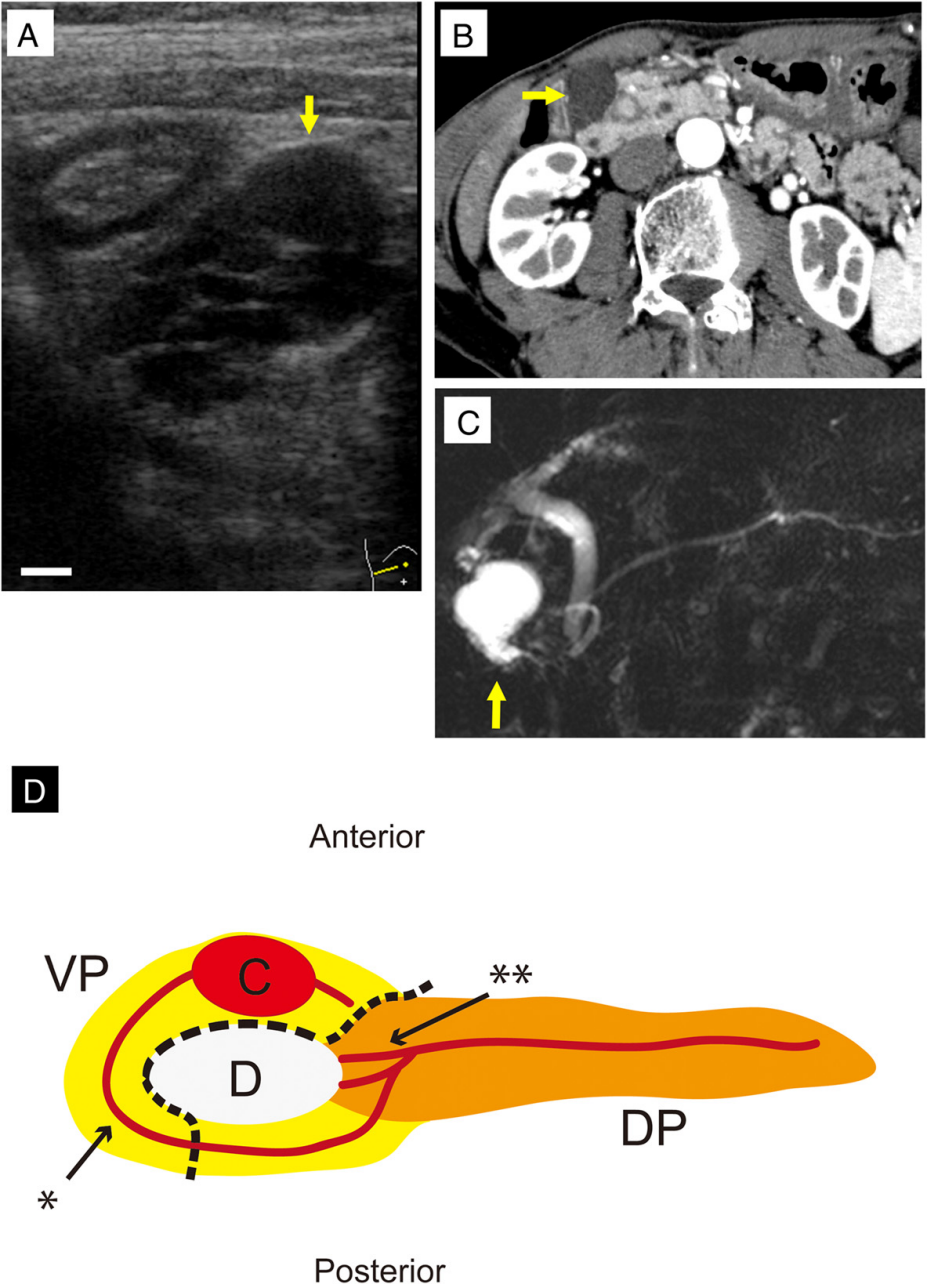
Diagnostic images of the intraductal papillary mucinous neoplasm (IPMN) in the patient’s annular pancreas. **a** A multilocular cystic mass was detected by abdominal ultrasonography (*arrow*). The cystic mass was 30 mm in diameter. No intramural nodules or calcifications were present in the cystic lesion. *Bar*, 5 mm. **b** Abdominal computed tomography revealed the presence of pancreatic tissue encircling the descending part of the duodenum (*arrow*). **c** Magnetic resonance cholangiopancreatography showed the annular duct encircling the duodenum. A 30-mm-diameter cystic mass was identified in the annular segment, and the mass was linked to the annular duct (*arrow*). The patient was preoperatively diagnosed with branch-duct-type IPMN (adenoma) with low-grade malignancy. **d** Schema of the annular pancreas in the present case. The *yellow region* indicates the ventral pancreas (*VP*). The *orange region* indicates the dorsal pancreas (*DP*). The *red lines* indicate the pancreatic ducts. The *dotted line* shows the resection line. *Single asterisk* shows the duct of Wirsung. *Double asterisks* show the duct of Santorini. *C* cystic mass, *D* duodenum

A 79-year-old Japanese man with epigastric pain was admitted to our hospital in January 2010. Abdominal ultrasonography revealed a large multilocular cystic mass (Fig. 1a). No intramural nodules or calcifications were present in the cystic lesion. Abdominal computed tomography showed that the pancreatic tissue encircled the descending part of the duodenum (Fig. 1b). A 30-mm-diameter cystic tumor was identified in the annular segment of the pancreas, and the tumor was linked to the annular duct. Magnetic resonance cholangiopancreatography also showed the annular duct encircling the duodenum (Fig. 1c), and the mass was linked to the main pancreatic duct. No intramural nodules or calcifications were detected in the tumor. The initial diagnosis was IPMN in the annular portion of the pancreas. These findings suggested a 30-mm-diameter branch-duct-type IPMN. Because the possibility of malignancy was not completely ruled out, we decided to remove the mass.

**Fig. 2:**
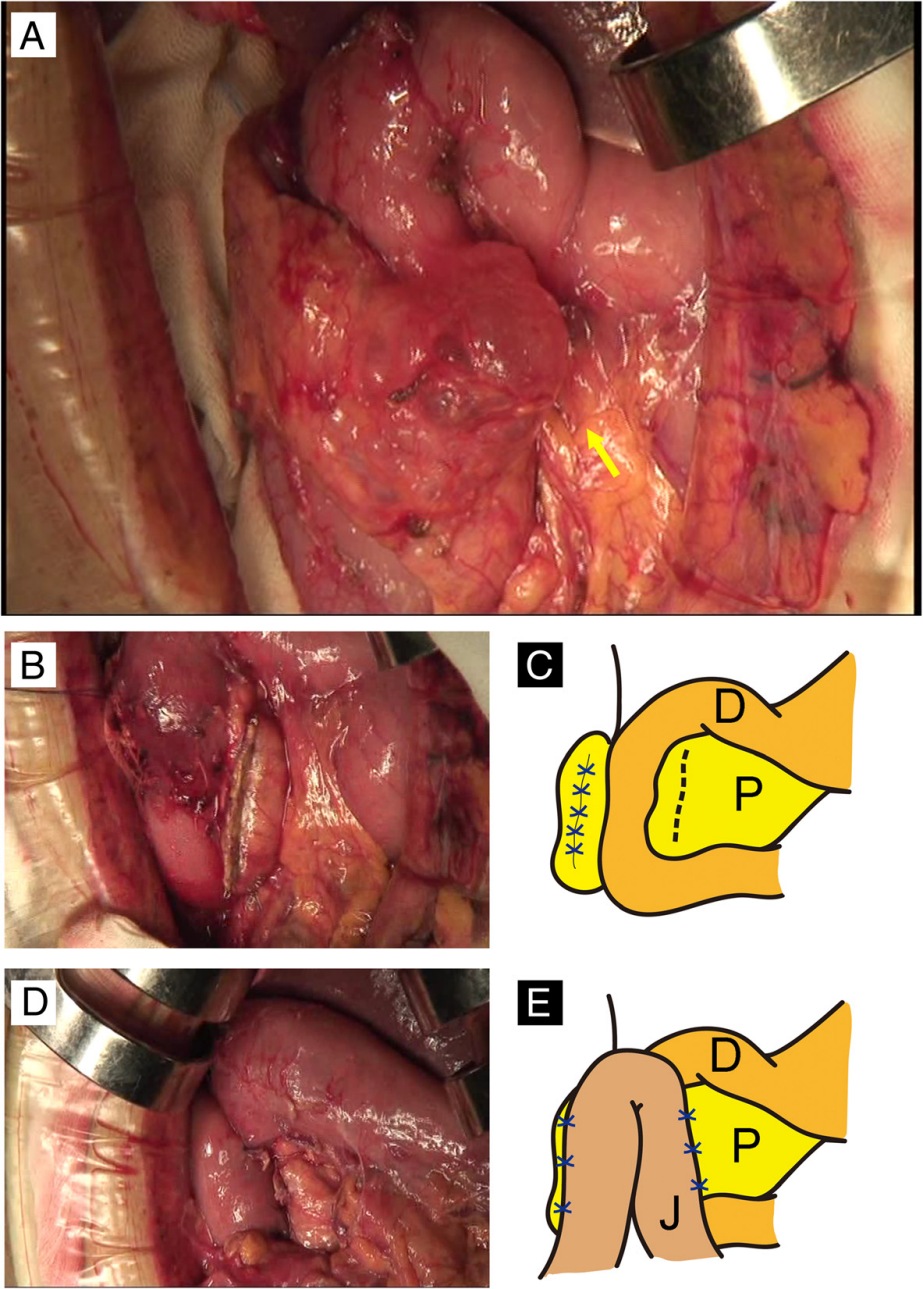
Limited resection of the annular segment and jejunal patch. **a** The pancreatic tissue encircling the descending part of the duodenum was detected intraoperatively. The cystic mass was also found in the annular segment. **b**, **c** The anterior annular segment was easily separated from the second portion of the duodenum. After resection of the annular segment, congestion of the separated surface of the duodenum was revealed. The schema of the appearance of the operative field after limited resection of the annular pancreas is demonstrated. **d**, **e** The second portion of the duodenum and the pancreatic stump were covered by the jejunum to prevent duodenal leakage and pancreatic fistula formation. The schema of the appearance of the operative field after this procedure is demonstrated. *D* duodenum, *P* pancreas, *J* jejunum

During surgery, we found that the pancreatic tissue encircled the second portion of the duodenum. The cystic mass was identified within the annular portion of the pancreas (Fig. 2a). Although loose adhesion of the annular portion of the pancreas to the duodenum was detected, the annular pancreas was carefully separated from the duodenum, preserving the serous membrane of the duodenum. All fibrotic bands between the duodenum and annular pancreas were ligated because small ducts between the duodenum and annular pancreas might be communicated with the pancreatic duct of the annular pancreas. Dissection of the annular portion (including the tumor) from the duodenum was completed without any injury to the duodenum. Frozen section examination of the surgical stump showed no malignancy. The distal side of the annular pancreas was dissected from the main pancreas using a linear endostapler (ENDO GIA Universal 60-mm length and 3.5-mm staples, Covidien). The pancreas parenchym was gently compressed by the endostapler while compressed for 3 min while the preserving pancreatic capsule, and then it was transected slowly. The proximal pancreatic duct stump was closed with 4-0 nonabsorbable sutures in a transfixing pattern, and the pancreatic tissue stump was closed with 3-0 nonabsorbable sutures in a vertical mattress pattern (Fig. 2b, c). Finally, the dissected surface of the duodenum and the cut surface of the pancreas were covered by the upper jejunal wall (Fig. 2d, e). The operation time was 310 min and intraoperative blood loss was 60 g. The patient’s postoperative course was uneventful, and he was discharged on postoperative day 22.

**Fig. 3:**
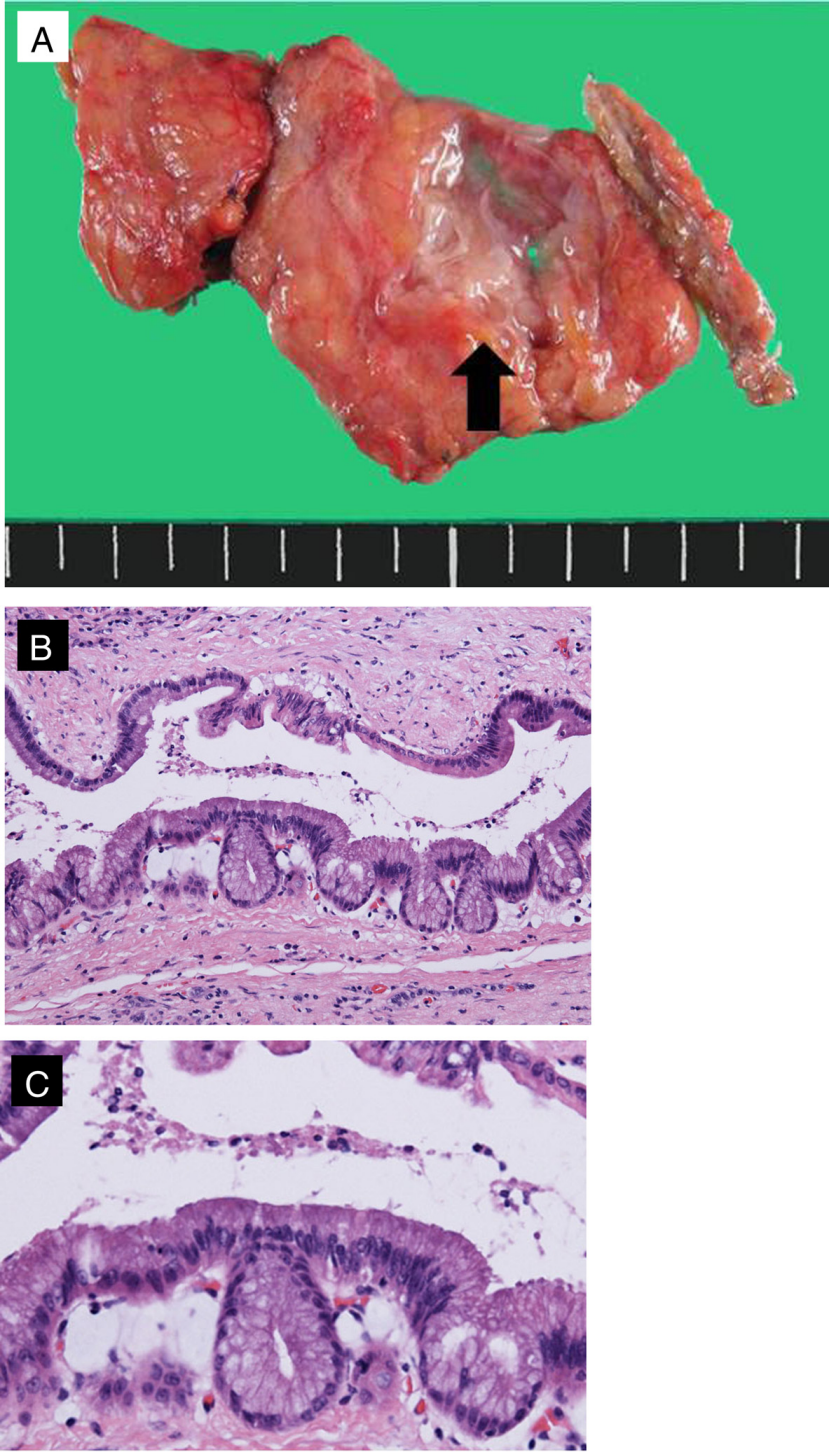
Histopathological findings. **a** The resected specimen showed dilatation of the annular pancreatic duct. The scale resolution shows 5 mm. Histopathological analysis showed a papillary architecture with nuclear atypia. **b** ×40. **c** ×200

The resected specimen contained the cystic tumor with dilatation of the annular pancreatic duct (Fig. 3a). Histopathological analysis revealed that the cystic tumor was derived from the dilated pancreatic duct. The ductal epithelium had a papillary architecture comprising mucin-rich columnar cells and high cellular density, but relatively low nuclear atypia (Fig. 3b, c). These findings were observed in both the main and branched pancreatic ducts. However, the surgical stump showed normal pancreatic tissue. Immunohistochemical analysis for p53 and Ki67 revealed less activity. Based on these results, the final diagnosis of the cystic tumor was an intraductal papillary mucinous adenoma with intermediated dysplasia in the annular pancreas. The patient had not developed recurrence 3 years after the resection.

### Discussion

Annular pancreas is a rare congenital anomaly in which a ring of pancreatic tissue encircles the second portion of the duodenum. Lecco reported that in patients with imperfect rotation of the ventral pancreatic anlage, a ring of pancreatic tissue might be left around the second portion of the duodenum [3]. Baldwin reported that a persistent left ventral bud of the pancreas causes it to encircle the duodenum [4]. The most commonly accepted theory explaining the development of an annular pancreas is that proposed by Lecco [3]. The annular pancreas has a bimodal pattern of presentation; the first peak occurs in infancy, and a later peak occurs in the fourth decade of life [5]. Combined congenital anomalies in children include trisomy 21, cardiac anomalies, and intestinal malrotation. Pediatric patients usually present with gastrointestinal obstruction and jaundice. In contrast, adults do not commonly present with annular pancreas, although it is being identified with increasing frequency because of more liberal performance of abdominal computed tomography, magnetic resonance cholangiopancreatography, and endoscopic retrograde cholangiopancreatography [5]. In this case, the patient presented with epigastralgia. The reason of epigastralgia was caused by repeated mild pancreatitis. The patient complained of mild epigastralgia and showed mild increase of CA19-9 and serum amylase levels in his first visit to our hospital. Then, in the second visit, the patient had no symptoms and those two data showed normal range. With respect to combined neoplasms, Zyromski et al. reported that neoplasms developed in 24 % of adults with an annular pancreas, and 48 % of them were pancreatobiliary tumors [5]. Systemic search of PubMed for publications in English was performed. Search terms were “annular pancreas,” “adult,” and “neoplasm.” Including our case, there are eight cases of pancreatic neoplasm associated with annular pancreas [6–12] (Table 1). The types of pancreatic neoplasm were as follows: four cases adenocarcinoma, two cases with mucinous cystic neoplasm, and one case with IPMN. The patients consisted of two males and six females with a mean age of 67.8 ± 14.5 years. The neoplasms occurred in three cases of the annular segment and five cases of the dorsal segment. Six patients underwent surgical resections of pancreatic neoplasm associated with annular pancreas, including pancreaticoduodenectomy in two cases, total pancreatectomy in one case, distal pancreatectomy in one case, and limited pancreatic resection in one case. An anomalous pancreatic duct could cause the stasis of pancreatic juice and gives inflammatory injury to pancreatic ductal epithelium. An exposure of such chronic stimulation may induce oncogenic change to the ductal epithelium. Thus, pancreatobiliary tumors in patients with an annular pancreas attribute to chronic inflammatory changes associated with pancreatitis [13, 14]. Thus, pancreatobiliary tumors in patients with an annular pancreas might be attributed to chronic inflammatory changes in this area associated with pancreatitis [15, 16]. The present patient had continuous epigastric pain, and a cystic neoplasm was found in the annular segment of the pancreas.

**Table 1:** The literature of pancreatic neoplasm associated with annular pancreas

Author	Year	Age	Gender	Origin	Pathology	Surgical treatment
Matsusue et al. [6]	1984	53	F	AP	Adenocarcinoma	Total pancreatectomy
Kamisawa et al. [8]	1995	79	F	DP	Adenocarcinoma	None
Yasui et al. [7]	1995	54	M	AP	Adenocarcinoma	Pylorus-preserving pancreaticoduodenectomy
Ben-David et al. [9]	2004	52	F	DP	Adenocarcinoma	Pancreaticoduodenectomy
Cholet et al. [10]	2004	88	F	DP	Adenocarcinoma	None
Ijichi et al. [11]	2009	60	F	DP	MCN	Pancreaticoduodenectomy
Milone et al. [12]	2013	78	F	DP	MCN	Distal pancreatectomy
Our case	2015	78	M	AP	IPMN	Limited pancreatic resection

*MCN* mucinous cystic neoplasm, *IPMN* intraductal papillary mucinous neoplasm, *AP* annular pancreas, *DP* dorsal pancreas

IPMNs are characterized by cystic dilation of the main and/or branched pancreatic ducts and intraductal proliferation of neoplastic mucinous cells arranged into papillary structures [17, 18]. These tumors have a wide spectrum of atypical grades ranging from low-grade dysplasia to invasive carcinoma [19]. The diagnosis of IPMN of the pancreas has markedly increased in the last few decades because of the widespread use of high-resolution imaging [20, 21]. IPMNs are classified as main duct type, branch duct type, and combined type, according to the area of involvement of the pancreatic ductal system [22, 23]. The present patient had a branch duct type of IPMNs (adenoma) with low-grade malignancy [22, 23]. However, the patient experienced abdominal pain, and the diameter of the tumor was large (30 mm). Although there is a controversy regarding whether pancreatic resection or close follow-up should be performed to treat IPMNs with low-grade malignant potential, especially in cases similar to the present case, we performed surgical extirpation of the tumor according to the Sendai consensus guidelines [21–26].

Various operative procedures are available to treat IPMNs with a low risk of malignancy [27–30]. Nakagohri et al. reported good surgical outcomes for noninvasive or minimally invasive IPMNs after inferior pancreatic head resection [31]. In this procedure, the uncinate process and pancreatic parenchyma around the duct of Wirsung are resected, preserving the pancreatic head around the duct of Santorini [31]. Takada described ventral pancreatectomy, which involves resection of only the ventral segment of the pancreas, preserving the dorsal segment and the main pancreatic duct [32]. Of course, division of the annular segment is generally not recommended because of the high incidence of postoperative complications such as fistula formation, pancreatitis, pancreatic laceration, and/or recurrent duodenal stenosis secondary to local fibrosis [5, 33, 34]. Thus, in the present case, the resection area was very carefully determined, and an additional procedure was performed to avoid postoperative complications.

In many cases, the pancreatic annulus cannot be separated because of the dense adhesion between the duodenum and annulus [2]. In our case, however, the adhesion between the annular segment and second portion of duodenum was fortunately loose, and we easily ligated the annular pancreatic duct. The annular segment (including the tumor) was removed, and the pancreatic head around the duct of Santorini was preserved. Additionally, the second portion of the duodenum and the pancreatic stump were covered by the jejunum to prevent duodenal leakage and pancreatic fistula formation. The present case suggests that limited annular pancreatic resection is safe when the adhesion between the duodenum and annular segment is loose and the locations of the annular pancreatic duct and the duct of Santorini are definitively identified. However, the precise indications for this procedure remain to be elucidated.

## Conclusions

In conclusion, the possibility of coexisting pancreatobiliary disorders such as IPMNs should be kept in mind in adult patients with an annular pancreas. Partial resection of the pancreas, including division of the annular segment, may be safe and effective in selected patients.

## Consent

Written informed consent was obtained from the patient for publication of this case report and any accompanying images.
